# Development of Low-Molecular-Weight Allosteric Agonist of Thyroid-Stimulating Hormone Receptor with Thyroidogenic Activity

**DOI:** 10.1134/S1607672922020016

**Published:** 2022-05-10

**Authors:** A. A. Bakhtyukov, K. V. Derkach, E. A. Fokina, V. N. Sorokoumov, I. O. Zakharova, L. V. Bayunova, A. O. Shpakov

**Affiliations:** 1 Sechenov Institute of Evolutionary Physiology and Biochemistry, Russian Academy of Sciences, St. Petersburg, Russia; 2 Institute of Chemistry, St. Petersburg State University, St. Petersburg, Russia

**Keywords:** receptor of thyroid-stimulating hormone, allosteric agonist, hypothyroidism, thyroid gland, thyroid hormone

## Abstract

To normalize the thyroid status in hypothyroidism caused by resistance to thyroid-stimulating hormone (TSH), low-molecular-weight allosteric agonists of TSH receptor can be used. A new compound ethyl-2-(4-(4-(5-amino-6-(*tert*-butylcarbamoyl)-2-(methylthio)thieno[2,3-d]-pyrimidine-4-yl)phenyl)-*1H*-1,2,3-triazol-1-yl) acetate (TPY3m), which stimulated the production of thyroxine when administered to rats (25 mg/kg, i.p.) and also increased the expression of thyroidogenic genes in the cultured FRTL-5 thyrocytes (30 μM) and the rat thyroid gland. The in vitro and in vivo treatment with TPY3m did not lead to a decrease in the expression of the TSH receptor gene in thyrocytes, restoring it under the conditions of receptor hyperactivation by the hormone. This determines the retaining and, in some cases, potentiation of the thyroidogenic effects of TSH (FRTL-5) or thyroliberin (rats) when they are coadministered with TPY3m. TPY3m is a prototype drug for correcting thyroid system functions in subclinical hypothyroidism.

The main regulator of the synthesis of thyroid hormones—thyroxine (T4) and triiodothyronine (T3)—in thyrocytes, specialized cells of the thyroid gland (TG), is thyroid-stimulating hormone (TSH), which is secreted by the adenohypophysis in response to stimulation with thyroliberin, hypothalamic TSH-releasing hormone (TRH) [1]. TSH binds specifically to the G-protein-coupled TSH receptor located on the surface of thyrocytes, thereby activating the adenylyl cyclase and phospholipase pathways and enhancing the expression and activity of proteins responsible for T4 synthesis (thyroglobulin, thyroperoxidase, and Na^+^/I^–^ cotransporter) and its conversion to T3 (D2-deiodinase) [2]. In the case of mutations in the ectodomain of the TSH receptor that prevent its binding to the hormone, as well as when the TSH receptor is exposed to inactivating autoantibodies, thyroid resistance to TSH develops, which leads to subclinical hypothyroidism [3, 4].

An approach to stimulate the TSH receptor may be the use of allosteric regulators with the activity of full agonists [5, 6]. Their characteristic feature is the ability to stimulate receptors of pituitary glycoprotein hormones that contain inactivating mutations in the ectodomain and are, therefore, insensitive to hormones [5]. Previously, we have developed peptide allosteric TSH receptor agonists capable of interacting with the allosteric site formed by cytoplasmic loops [7]. However, although they were active in vitro, their activity in vivo was limited due to their degradation. A more promising approach is to develop more stable heterocyclic compounds that can penetrate into the allosteric site located in the transmembrane domain of the TSH receptor [5]. On the basis of the structure of thieno[2,3-d]-pyrimidine, we have developed the compound TP48, which exhibits the activity of an allosteric antagonist of the TSH receptor and can be used to normalize the thyroid status in autoimmune hyperthyroidism [8]. The aim of this study was to create a ligand for the allosteric site of the TSH receptor, which, similarly to TP48, has a thieno[2,3-d]-pyrimidine structure but, in contrast to TP48, activates the TSH receptor. The task was to study its effect on the gene expression of thyroidogenic proteins and the TSH receptor in thyrocyte culture and to investigate its effect on basal and TRH-stimulated thyroidogenesis in rats in vivo.

To synthesize ethyl-2-(4-(4-(5-amino-6-(*tert*-butylcarbamoyl)-2-(methylthio)thieno[2,3-d]-pyrimidin-4-yl)phenyl)-*1H*-1,2,3-triazol-1-yl) acetate (TPY3m), we used the reaction between 5-amino-*N*-(*tert*-butyl)-4-(4-ethynylphenyl)-2-(methylthio)thieno[2,3-d]-pyrimidine-6-carboxamide and ethyl-2-azidoacetic acid in the presence of copper-containing catalysts. After HPLC purification, the resulting product (mp. 218.1–218.6°C) was characterized by ^1^H-NMR (Bruker Avance III 400 instrument, Germany) and mass spectrometry (Bruker micrOTOF mass spectrometer, Germany). The ^1^H-NMR spectrum (400 MHz, chloroform-d): δ 8.09–8.03 (m, 3H Ar phenyl + Ar triazol), 7.76 (d, *J* = 7.9 Hz, 2H Ar phenyl), 5.32 (s, 2H CArCH_2_C(O)), 5.26 (m, 3H NH_2_ + NH), 4.34 (q, *J* = 7.1 Hz, 2H CH_2_ C(O)OEt), 2.69 (s, 3H MeS), 1.48 (s, 9H *t*Bu), 1.36 (t, *J* = 7.2 Hz, 3H CH_3_ C(O)OEt). Mass spectrum (ESI+, 100 V, CH_3_OH): found, 526.1695 [M+H]; calculated for C_24_H_28_N_7_O_3_${\text{S}}_{2}^{ + }$, 526.1690.

In the experiments, we used 3-4-month-old Wistar rats, which were housed under standard vivarium conditions with an ad libitum access to food and water. The FRTL-5 thyrocyte cell line was obtained from the European Collection of Authenticated Cell Cultures. The cells were cultured in F-12 medium supplemented with 6 hormones and growth factors (F-12+6Н): 1 IU/mL TSH (Elabscience, United States), 10 μg/mL insulin, 5 μg/mL liver growth factor, 10 nM hydrocortisone (Sigma, United States), 10 ng/mL somatostatin (Tocris Bioscience, United Kingdom), and 5 µg/mL transferrin (Biolot, Russia) [9]. Before the experiment, the cells were removed from the substrate with a trypsin–versene (1 : 1) solution, subcultured into the complete growth medium F-12+6H (4.3 × 10^4^ cells/0.25 mL of medium per well) for 48 h, and then transferred to the F-12+5H medium without TSH. After 24 h of incubation, the cells were incubated with 30 μM TPY3m, 6 mIU/mL TSH, or TPY3m+TSH. After 6 h of incubation, the expression of the target genes was assessed.

In in vivo experiments, TPY3m was administered to rats (25 mg/kg, ip, in 200 µL DMSO). TRH (Sigma, United States) was administered intranasally at a dose of 100 µg per rat as described previously [10]. Control rats received DMSO instead of TPY3m and saline instead of TRH. TRH was administered 30 min after TPY3m. Four groups were formed (*n* = 5 in all groups): control and groups with TPY3m, TRH, and TPY3m+TRH treatment. Blood samples were collected from the tail vein using anesthesia with 2% lidocaine solution before and 1.5 and 3 h after TRH administration. The levels of free (fT4) and total (tT4) thyroxine and free (fT3) and total (tT3) triiodothyronine were determined using reagent kits from Immunotech (Russia).

Gene expression in FRTL-5 cells and thyroid tissue was assessed by real-time PCR. For this purpose, total RNA was isolated using the Extract RNA kit, and reverse transcription was performed using the MMLV RT Kit (Evrogen, Russia). The amplification mixture contained 10 ng of the PCR product, 0.4 µM each of the forward and reverse primers, and the qPCRmix-HS SYBR+LowROX reagent (Evrogen, Russia). The signal was detected using a 7500 Real-Time PCR System amplifier (Thermo Fisher Scientific Inc., United States). Data were calculated by the delta-delta C_t_ method using *18S rRNA* (18S-rRNA) and *Actb* (β-actin) as reference genes. Expression of thyroglobulin (*Tg*), thyroperoxidase (*TPO*), Na^+^/I^–^ cotransporter (*Nis*), D2-deiodinase (*Dio2*), and TSH receptor (*TshR*) was analyzed.

Statistical analysis of the results was performed using the Microsoft Office Excel 2007 software; the normality of distribution was tested using the Shapiro–Wilk test. To compare two samples with a normal distribution, Student’s *t* test was used; four groups were compared using Tukey’s correction analysis of variance. Data were presented as *M* ± *SEM*; differences were considered significant at *p* < 0.05.

The addition of TSH and TPY3m to the incubation medium after 24-h TSH deprivation led to an increase in the *Nis* gene expression in FRTL-5 cells, with the effect of TSH being more pronounced (Fig. 1). TSH also increased the expression of the *Tg* gene (Fig. 1). In the case of a combined administration of TSH and TPY3m, an increase in the stimulatory effect of TSH on the expression of the *Tg* gene was observed, and the effect of the hormone on the *Nis* gene expression was retained (Fig. 1). Thus, TPY3m exhibits the properties of a TSH receptor agonist and does not prevent the effects of TSH. This is due to different localization of the orthosteric site to which TSH binds (ectodomain) and the allosteric site to which low-molecular-weight regulators of the receptor bind (transmembrane domain) [11]. TPY3m had little effect on *TshR* expression, whereas TSH decreased it, which was due to the compensatory response of cells to TSH-induced receptor hyperactivation and was previously shown by M. Saji еt al. [12]. In the case of a combined administration, partial restoration of the expression of the TSH receptor gene was detected (Fig. 1).

**Fig. 1.**
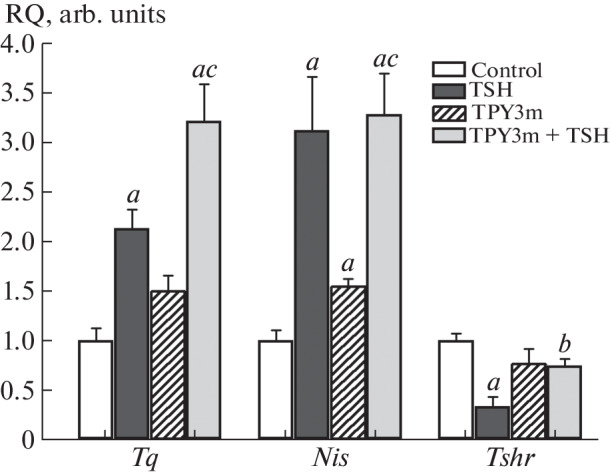
Effects of TSH and TPY3m on the expression of thyroglobulin (*Tg*), Na^+^/I^–^ cotransporter (*Nis*), and TSH receptor (*TshR*) genes in FRTL-5 thyrocyte culture preincubated in TSH-free medium. Designations: TSH, TPY3m, and TPY3m + TSH—cells incubated with TSH (6 mIU/mL), TPY3m (30 µM), and TSH and TPY3m together. Differences with C (^a^), with TSH (^b^), and between TPY3m and TPY3m+TSH (^c^) are statistically significant at *p* < 0.05. Data are presented as *M* ± *SEM*, *n* = 5.

Intraperitoneal administration of TPY3m to rats after 2 h increased the level of tT4, and after 3.5 h the levels of both forms of T4 increased (Table 1). TRH was more active and increased the levels of all thyroid hormones after 3 h (Table 1).

**Table 1.** The stimulating effects of thyroliberin and TPY3m in combined and separate use on the levels of thyroid hormones in the blood of rats

Group	fT4, pM	tT4, nM	fT3, pM	tT3, nM
2 h after administration of TPY3m and 1.5 h after administration of TRH				
Control	24.32 ± 1.51	47.86 ± 1.64	2.37 ± 0.13	2.59 ± 0.15
TRH	37.04 ±0.75^a^	68.66±3.16^a^	3.77±0.21^a^	3.23 ± 0.35
TPY3m	28.26 ± 1.54^b^	55.54±1.39^a,b^	2.78 ± 0.14^b^	2.89 ± 0.10
TPY3m+TRH	38.02 ± 0.97^a,c^	75.48±1.72^a,c^	4.02 ±0.16^a,c^	3.34 ± 0.21
3.5 h after administration of TPY3m and 3 h after administration of TRH				
Control	23.00 ± 0.55	46.30 ± 1.04	2.28 ± 0.12	2.35 ± 0.11
TRH	33.06 ±1.30^a^	69.02 ±1.29^a^	3.30 ±0.19^a^	3.02 ±0.13^a^
TPY3m	28.76±0.67^a,b^	57.50 ±3.08^a,b^	2.82 ± 0.13	2.75 ± 0.17
TPY3m+TRH	39.04 ±0.67^a,b,c^	81.10±1.31^a,b,c^	4.94±0.24^a,b,c^	3.69±0.13^a,b,c^

TPY3m (the TPY3m and TPY3m+TRH groups) was administered at a dose of 25 mg/kg (ip), and TRH (TRH and TPY3m+TRH groups) was administered 30 min later at a dose of 100 μg per rat (intranasally). The differences with the control (a) and with the TRH group (b) and between the TPY3m and TPY3m+TRH groups (c) are statistically significant at *p* < 0.05. Data are presented as *M* ± *SEM*, *n* = 5.

In the case of combined administration of TRH and TPY3m, the stimulation of the production of thyroid hormones increased. In 3.5 h, the increase in the level of both forms of T3 was greater than the sum of the increases after the administration of TRH or TPY3m, which may indicate a potentiating effect of TPY3m on the TRH-induced stimulation of fT3 and tT3 production (Table 1). The content of total and free T4 at a combined administration of TRH or TPY3m was higher than at their separate administration; however, no potentiating effect was found in this case (Table 1). Thus, by its ability to stimulate the production of thyroid hormones in rats, TPY3m can be classified as an allosteric TSH receptor agonist.

The study of gene expression in the thyroid gland showed that TRH increases the expression of the *Tg*, *TPO*, and *Dio2* genes and reduces the expression of the TSH receptor gene (Fig. 2). TPY3m increased the expression of the *TPO* and *Dio2* genes. At the same time, the expression of the *TshR* gene in the TPY3m treatment group not only did not decrease but even increased, although the differences with the control were not significant (Fig. 2). It should be noted that thieno[2,3-d]-pyrimidines with the activity of agonists of the luteinizing hormone receptor, related to the TSH receptor, also have little effect on the expression of their receptor [13]. Under conditions of combined administration, the stimulatory effects of TRH on the expression of thyroidogenic genes were retained, and in the case of the *Nis* gene the effect of TRH was enhanced (Fig. 2). Along with this, the inhibitory effect of TRH on the *TshR* gene expression disappeared (Fig. 2). We assumed that the restoration of *TshR* expression in the TPY3m+TRH group compared to the TRH group ensures normalization of thyrocyte sensitivity to TSH receptor agonists. Partial restoration of the *TshR* expression in the presence of TPY3m was also observed by us in thyrocyte culture (Fig. 1). Thus, the potentiating effect of TPY3m on *Tg* expression in the FRTL-5 thyrocyte culture and on T3 production and *Nis* expression in the thyroid gland of rats of the TPY3m + TRH group may be, at least in part, due to normalization of the TSH receptor expression and retaining of the activity of TSH-dependent cascades under conditions of their hyperactivation with TSH. However, it cannot be ruled out that TPY3m may not only possess agonist activity but also function as a positive allosteric modulator (PAM). Such activity is exhibited by a number of ligands of the allosteric site of G-protein-coupled receptors, referred to as ago-PAM [14, 15].

**Fig. 2.**
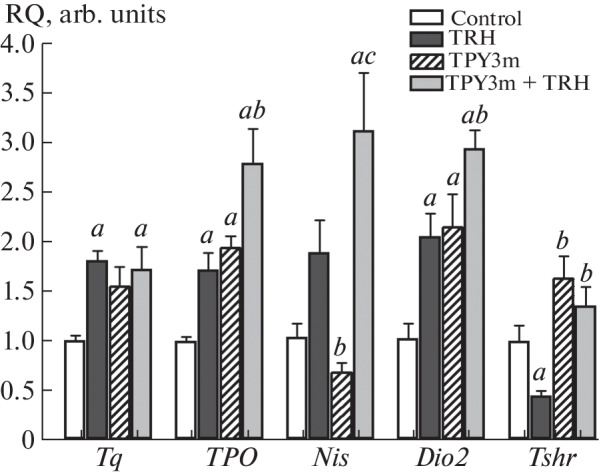
Effects of thyroliberin and TPY3m on the expression of genes encoding thyroidogenic proteins and TSH receptor in rat thyroid gland. Designations: C—control; TRH, TPY3m and TPY3m+TRH—the groups of rats treated with TRH (100 μg/rat, intranasally), TPY3m (25 mg/kg, i.p.) and with TRH + TPY3m. The differences with the control (*a*) and with the TRH group (*b*) and between the TPY3m and TPY3m+TRH groups (*c*) are statistically significant at *p* < 0.05. *M* ± *SEM*, *n* = 5.

Thus, on the basis on the thieno[2,3-d]-pyrimidine structure, we have developed a new allosteric agonist of the TSH receptor, TPY3m, which stimulated the production of thyroxin when administered intraperitoneally to rats and also stimulated the expression of thyroidogenic genes in the thyrocyte cell culture FRTL-5 and in rat thyroid gland. TPY3m caused no decrease in the expression of the TSH receptor gene and, when combined with TSH (FRTL-5) or TRH (TG), restored it. This, in our opinion, determines the retaining and, in some cases, potentiation of the effects of TSH (FRTL-5) or TRH (rats) on thyroidogenesis. TPY3m can be considered as a prototype drug for correcting the thyroidogenic function of the thyroid gland in subclinical hypothyroidism, including that caused by resistance to TSH, as well as for accelerating the uptake of radioactive iodine by thyrocytes in diagnosing and radiotherapy of thyroid cancer.
